# Role of *IL1A* rs1800587, *IL1B* rs1143627 and *IL1RN* rs2234677 Genotype Regarding Development of Chronic Lumbar Radicular Pain; a Prospective One-Year Study

**DOI:** 10.1371/journal.pone.0107301

**Published:** 2014-09-10

**Authors:** Aurora Moen, Elina Iordanova Schistad, Lars Jørgen Rygh, Cecilie Røe, Johannes Gjerstad

**Affiliations:** 1 National Institute of Occupational Health, Oslo, Norway; 2 Department of Physical Medicine and Rehabilitation, Oslo University Hospital, Ullevaal, Oslo, Norway; 3 Faculty of Medicine, University of Oslo, Oslo, Norway; 4 Department of Anesthesiology and Intensive Care, Haukeland University Hospital, Bergen, Norway; 5 Department of Molecular Biosciences, University of Oslo, Oslo, Norway; University of Texas at Dallas, United States of America

## Abstract

Previous studies indicate that lumbar radicular pain following disc herniation may be associated with release of several pro-inflammatory mediators, including interleukin-1 (IL1). In the present study, we examined how genetic variability in *IL1A* (rs1800587 C>T), *IL1B* (rs1143627 T>C) and *IL1RN* (rs2234677 G>A) influenced the clinical outcome the first year after disc herniation. Patients (n = 258) with lumbar radicular pain due to disc herniation were recruited from two hospitals in Norway. Pain and disability were measured by visual analogue scale (VAS) and Oswestry Disability Index (ODI) over a 12 month period. The result showed that patients with the *IL1A* T allele, in combination with the *IL1RN* A allele had more pain and a slower recovery than other patients (VAS *p* = 0.049, ODI *p* = 0.059 rmANOVA; VAS *p* = 0.003, ODI *p* = 0.050 one-way ANOVA at 12 months). However, regarding the *IL1B*/*IL1RN* genotype, no clear effect on recovery was observed (VAS *p* = 0.175, ODI *p* = 0.055 rmANOVA; VAS *p* = 0.105, ODI *p* = 0.214 one-way ANOVA at 12 months). The data suggest that the *IL1A* T/*IL1RN* A genotype, but not the *IL1B* T/*IL1RN* A genotype, may increase the risk of a chronic outcome in patients following disc herniation.

## Introduction

Studies of patients with lumbar disc herniation verified by MRI suggest that mechanical compression of the nerve-roots may induce lumbar radicular pain. However, disc herniation is also associated with an inflammatory response, with increased levels of pro-inflammatory mediators, such as interleukins (ILs), matrix metalloproteinases (MMPs) and prostaglandines (PGs) [1], [2]. These inflammatory agents may promote disc degeneration, as well as sensitize the primary afferent sensory fibers.

Several genetic variants may affect such processes and influence pain sensitivity. These might involve genetic variants important for the expression of GTP cyclohydrolase close to the nerve roots [3], MMP1 in the intervertebral disc [4] or genetic variants in genes relevant to immunological systemic responses such as HLA [5]. In addition, genetic variability in *IL1A* may reduce the pain threshold after disc herniation [6].

The IL1 gene family includes IL1α and IL1β, as well as the interleukin-1 receptor antagonist (IL1Ra). The former two are strong inducers of inflammation and one of the first cytokines produced by stress. They are secreted by a variety of cells, including monocytes, endothelial cells and disc cells [1], [7]. The actions of these cytokines are, however, balanced by the expression of the endogenous IL1Ra (encoded by *IL1RN*). This naturally occurring antagonist binds to IL1 receptors in competition with IL1, but does not elicit an intracellular response upon binding.

IL1α, IL1β and IL1Ra and their composite genotype have previously been linked to disc degeneration and low back pain in the general population [8], [9]. We hypothesized that patients with a high expression of IL1α (carriers of rs1800587 T allele) or high expression of IL1β (carriers of rs1143627 T allele), combined with a reduced expression of IL1Ra (carriers of rs2234677 A allele), would have an increased risk of long-lasting clinical pain as well. Hence, the purpose of our study was to investigate the combined effect *IL1A,* or *IL1B,* and *IL1RN* genotypes in recovery after disc herniation.

## Materials and Methods

### Patients

Patients aged 18–60 years, with confirmed lumbar disc herniation by magnetic resonance imaging (MRI) with corresponding sciatic pain and positive Straight Leg Raising (SLR) test were recruited from Oslo University Hospital, Ullevaal, Norway and Haukeland University Hospital, Norway, for details see [10]. A total of 258 patients, all European-Caucasians referred to the hospitals in 2007–2009, were included. However, at inclusion, 6 patients changed their mind and did not want to participate, which gave data from 252 patients. In addition, 21 patients (8%) were lost during the follow-up.

### Ethics

All participants received written information and signed an informed consent form. The study was approved by the Norwegian Regional Committee for Medical Research Ethics and the Norwegian Social Science Data Services.

### Clinical procedure

After inclusion, the patients were followed up at 6 weeks, 6 months and 12 months. Conservative treatment was received by 42%, while the remaining 58% received surgery. At inclusion all patients underwent a standardized clinical examination with assessment of sensory and motor function and tendon reflexes at the lower limbs as well as an MRI scan. At 6 weeks and 12 months follow-up the clinical examination was repeated, while at 6 months follow-up patients reported their condition by a telephone interview and answered questionnaires by mail.

All patients were asked to rate their pain intensity in activity during the last week on a 10-cm visual analogue scale (VAS) with endpoints “no pain” and “worst possible pain”. The validated Norwegian version of the Oswestry Disability Index (ODI) was used to assess problems with physical function related to low back pain [11]. The sampling of the clinical data was completed before the genotyping of the patients was performed.

### Genotyping

Genomic DNA was extracted from whole blood cells using FlexiGene DNA isolation kit (Qiagen, Hilden, Germany). SNP genotyping was carried out using predesigned TaqMan SNP genotyping assays (Applied Biosystems) for *IL1A* rs1800587 C>T, *IL1B* rs1143627 T>C and *IL1RN* rs2234677 G>A. Approximately 10 ng genomic DNA was amplified in a 5 µl reaction mixture in a 384-well plate containing 1x universal TaqMan master mix and 1x assay mix, the latter containing the respective primers and MGB-probes. The probes were labeled with the reporter dyes FAM or VIC at 5′end to distinguish between the two alleles. The reactions were performed on an ABI 7900HT sequence detection system (Applied biosystems) at the following program: After initial denaturation and enzyme activation at 95°C for 10 min, the reaction mixture was subjected to 60 at 95°C for 15 s and 60°C for 1 min. Negative controls containing water instead of DNA were included in every run. Genotypes were determined using the SDS 2.2 software (Applied Biosystems). Approximately 10% of the samples were re-genotyped and the concordance rate was 100%.

### Statistics

VAS activity score and ODI measurements over time were compared regarding genotypes by repeated measures analysis of variance (rmANOVA). When sphericity assumption was not met, a Greenhouse-Geisser correction was applied. Separate analyses were performed to check for potential confounding effects of the covariates age, gender, treatment and smoking status. Covariates with a p-value less than 0.1 were kept in the final model (Table 1). Further, VAS activity score and ODI score at 12 months after disc herniation were examined regarding genotypes by one-way ANOVA and Tuckey honestly significance difference (HSD) post-hoc comparisons.

**Table 1:** Significance of covariates.

Repeated measures ANOVA							
		*IL1A* and *IL1RN*			*IL1B* and *IL1RN*		
Outcomemeasure	Covariates	WithinSubjectseffects.*p*-values	BetweenSubjectseffects.*p*-values	Included infinal model.yes/no	WithinSubjectseffects.*p*-values	BetweenSubjectseffects.*p*-values	Included infinal model.yes/no
**VAS**	Age	0.683	0.795	No	0.654	0.528	No
	Gender	0.243	0.355	No	0.422	0.561	No
	Smoking	0.947	0.050	Yes	0.955	0.002	Yes
	Treatment	0.000	0.515	Yes	0.000	0.594	Yes
**ODI**	Age	0.145	0.091	Yes	0.170	0.054	Yes
	Gender	0.473	0.114	No	0.799	0.185	No
	Smoking	0.836	0.006	Yes	0.731	0.003	Yes
	Treatment	0.000	0.639	Yes	0.000	0.692	Yes

The table gives an overview between covariates and the three outcome measures: VAS and ODI. Covariates with a *p* value≤0.1 were included in the final model.

The combined effect of *IL1A* or *IL1B*, and *IL1RN* genotypes were analyzed. *IL1A* C/T and *IL1RN* G/A genotypes were grouped in the following variables: 1) *IL1A* C/C and *IL1RN* G/G, 2) *IL1A* */T or *IL1RN* */C, and 3) *IL1A* */T and *IL1RN* */C. *IL1B* T/C and *IL1RN* G/A genotypes were grouped in the following variables: 1) IL1β C/C and *IL1RN* G/G, 2) *IL1B* */T or *IL1RN* */C, and 3) *IL1B* */T and *IL1RN* */C. Statistical analyses were performed by using the SPSS (version 20) statistical package. A p-value less than 0.05 was set as the level of statistical significance. The data are shown as mean ± SEM.

## Results

**Figure 1:**
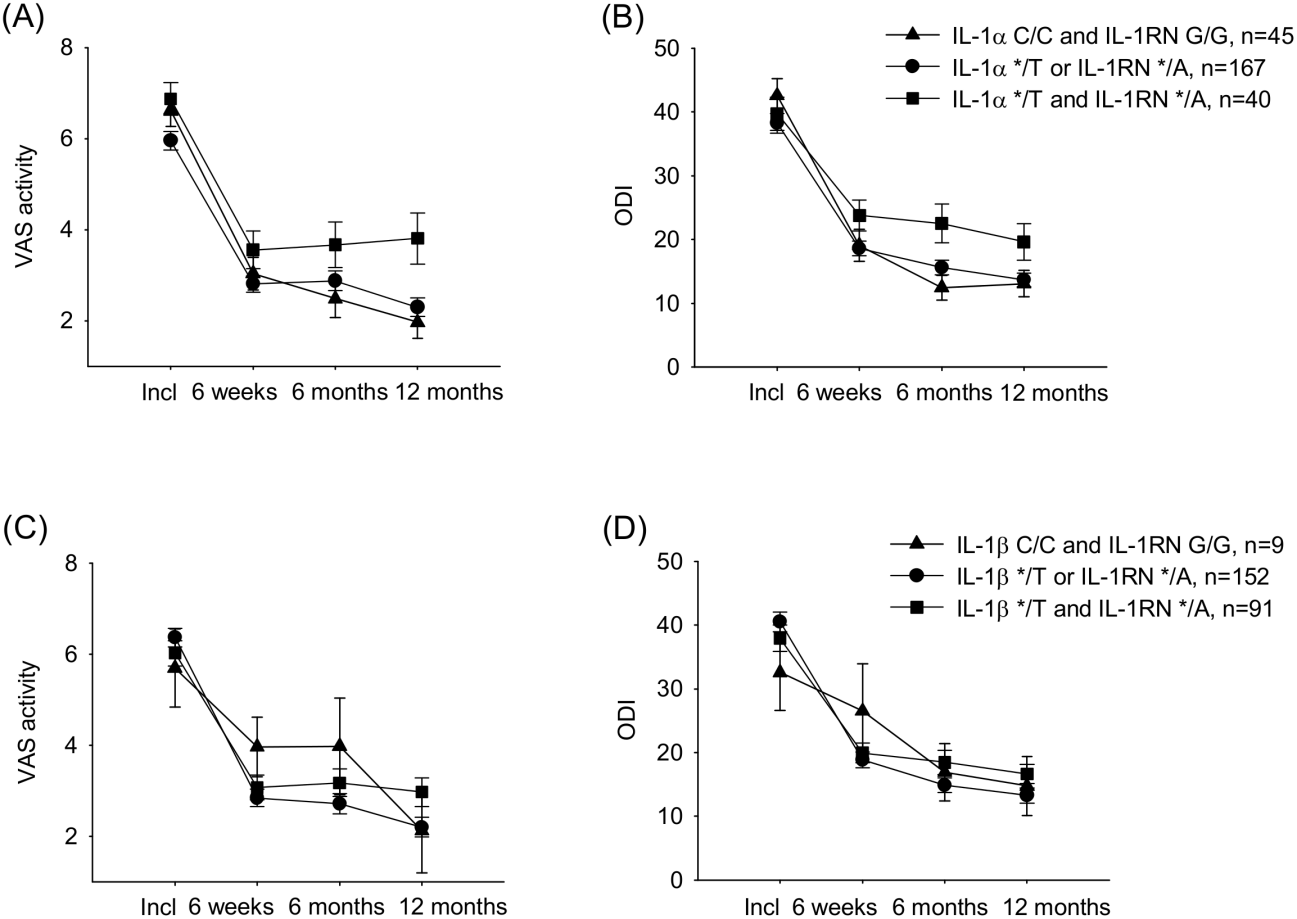
The time course of clinical outcome measures following disc herniation. A) and B) patients grouped by *IL1A* C/T and *IL1RN* G/A genotypes. VAS activity score (*p* = 0.049 rmANOVA; *p* = 0.003 one-way ANOVA at 12 months), ODI score (*p* = 0.059 rmANOVA; *p* = 0.050 one-way ANOVA at 12 months). C) and D) patients grouped by *IL1B* T/C and *IL1RN G/A* genotypes. VAS activity score (*p* = 0.175 rm ANOVA; *p* = 0.105 one-way ANOVA at 12 months), ODI score (*p* = 0.055 rmANOVA; *p* = 0.214 one-way ANOVA at 12 months). Data are shown as means ± SEM.

The analysis of clinical outcome over time, i.e. from inclusion to 12 months, revealed that the progression of pain and disability after lumbar disc herniation may be associated with the *IL1A*/*IL1RN* genotype. In accordance with our hypothesis, patients with both rare alleles (*IL1A* */T and *IL1RN* */A) seemed to report more pain and have a slower recovery than other patients (Figure 1 A, B). A significant association between genotype and VAS activity score, and a borderline significant association regarding ODI score, were observed (VAS score *p* = 0.049, ODI score *p* = 0.059 within-subjects effects rm ANOVA). No clear association were, however, found for the clinical outcomes, with regard to the *IL1B*/*IL1RN* genotype (Figure 1 C, D; VAS score *p* = 0.175, ODI score *p* = 0.055 within-subjects effects rm ANOVA). The characteristics of the cohort of patients stratified by the *IL1A/IL1RN* genotype and the *IL1B/IL1RN* genotype are listed in Table 2.

**Table 2:** Characteristics of patients grouped by the *IL1A*/*IL1B* and *IL1RN* genotypes.

	Gender,men/women (%)	Mean age(min/max)	Current smoker,yes/no (%)	Treatment,surgery/conservative (%)
***IL1A*** ** C/T and ** ***IL1RN*** ** G/A**				
* IL1A* C/C andIL1RN G/G, n = 45	20/25 (44/56)	42 (22–59)	12/33 (27/73)	27/18 (60/40)
* IL1A* T/* or IL1RNA/*, n = 167	91/76 (54/46)	40 (18–60)	61/106 (37/63)	98/69 (59/41)
* IL1A* T/* andIL1RN A/*, n = 40	24/16 (60/40)	43 (25–59)	19/21 (48/52)	21/19 (52/48)
***IL1B*** ** T/C and ** ***IL1RN*** ** G/A**				
* IL1B* C/C andIL1RN G/G, n = 9	4/5 (44/56)	44 (32/60)	3/6 (33/67)	2/7 (22/78)
* IL1B* T/* or IL1RNA/*, n = 152	85/67 (56/44)	41 (18/60)	54/98 (36/64)	96/56 (63/37)
* IL1B* T/C andIL1RN A/*, n = 91	46/45 (51/49)	41 (19/59)	35/56 (38/62)	48/43 (53/47)

Min, minimum; max, maximum.

Further analyses of the data sampled at 12 months showed that patients with the *IL1A* */T and *IL1RN* */A genotype had an increased one-year risk of a negative outcome, with more pain and poorer recovery (VAS score *p* = 0.003, ODI *p* = 0.050, One-way ANOVA). Post-hoc comparisons confirmed the differences between carriers of both *IL1A* T and *IL1RN* A alleles compared to none-carriers at 12 months (VAS score *p* = 0.006, ODI score *p* = 0.051, Tuckey HSD post-hoc comparisons*)*. In contrast, no significant influence of the *IL1B/IL1RN* genotype on the one-year outcome was observed (VAS score *p* = 0.105, ODI *p* = 0.214, One-way ANOVA). Mean ± SEM values at 12 months are listed in Table 3.

**Table 3:** Pain- and disability ratings at 12 months.

	VAS	ODI
***IL1A*** ** C/T and ** ***IL1RN*** ** G/A**		
* IL1A* C/C and *IL1RN* G/G	1.97±0.36	13.10±2.08
* IL1A* T/* or *IL1RN* A/*	2.30±0.20	13.68±1.05
* IL1A* T/* and *IL1RN* A/*	3.81±0.56	19.68±2.86
***IL1B*** ** T/C and ** ***IL1RN*** ** G/A**		
I*L1B* C/C and *IL1RN* G/G	2.13±0.93	14.75±4.63
* IL1B* T/* or *IL1RN* A/*	2.20±0.21	13.25±1.17
* IL1B* T/* and *IL1RN* A/*	2.97±0.31	16.63±1.52

The table shows the 12 months VAS and ODI scores for the patients grouped by the combinations of *IL1A* C/T, *IL1B* T/C and *IL1RN* G/A genotypes. Mean ± SEM values are shown.

## Discussion

In the present study we investigated the clinical impact of the genetic variants *IL1A* rs1800587 C>T, *IL1B* rs1143627 T>C and *IL1RN* rs2234677 G>A in patients following disc herniation. As expected, all patients suffered severe pain when they first were referred to the hospitals. However, patients carrying the *IL1A* T allele in combination with the *IL1RN* A allele (both rare alleles of these SNPs) reported more pain and disability during the follow-up period than the patients carrying only one or neither of these alleles. In line with previous data obtained from 131 occupationally active Finish men [8], our findings suggest that radicular low back pain may be dependent upon the *IL1A* T/*IL1RN* A genotype.

The *IL1A* T allele has been associated with an enhanced promoter activity resulting in increased gene expression, both at mRNA and at protein level, compared to the C allele [12]. Moreover, earlier data suggest that the *IL1RN* A allele, since it is in linkage disequilibrium with a VNTR polymorphism in intron 2 of the *IL1RN* gene [13], [14], is associated with reduced in vitro monocyte IL1Ra synthesis and enhanced IL1β production [15], [16]. Thus, it seems likely that patients with the *IL1A* T/*IL1RN* A genotype have overall enhanced levels of IL1 relative to IL1Ra, resulting in an increased activation of IL1 receptors and a more pronounced inflammatory response.

Analyses of intervertebral disc samples from patients suggest that a high IL1 to IL1Ra ratio may be associated with degenerative disc disease [17]. IL1 may induce up-regulation of matrix-degrading enzymes, such as MMPs, and inhibit resynthesis of proteoglycans [17]–[19], thereby promoting degradation of the intervertebral disc matrix. Moreover, *IL1A* together with *MMP3* gene variation has previously been associated with Modic changes [20]. Genetic variability in *MMP1* has also been suggested to contribute to low back pain and sciatica [4].

IL1, along with IL6 and TNF, secreted by a herniated disc may sensitize peripheral nociceptors. For example, IL1α increases the production of PGE2 in human disc cells in a dose dependent manner [2], and alter the expression of substance P in rat DRG neurons [21]. IL1α is also active as a precursor. Peripheral and central administration of IL1Ra has been found to alleviate inflammatory hyperalgesia in mice [22]. Hence, IL1 may indirectly facilitate nociceptive transmission, which may explain the increased pain experience reported by patients with the *IL1A T/IL1RN A* genotype.

Regarding the *IL1B* T/C SNP, it has been suggested that a shift from T to C results in a disruption of the TATA box resulting in a less active promoter. The T allele confers higher expression of the IL1β gene compared to the C allele [23]. Actually, *IL1B* polymorphisms have previously been associated with symptomatic lumbar disc herniation [24], and together with *IL1RN* polymorphism associated with low back pain in the general population [8]. In the present study, however, we did not find any associations between clinical pain and the *IL1B* T/*IL1RN* A genotype.

In addition to genetic influences, other risk factors such as physical work factors, psychosocial aspects and smoking may contribute to development of persistent back pain and disability [25]–[27]. Hence, the link between the IL1 genotype and persistent pain may be related to an increased inflammatory response, but also to more complex mechanisms. For example, the effect of genotype may be influenced by environmental factors [28]. Moreover, a gene - lifestyle interaction is possible [29]. Our data suggested a possible association between smoking and persistent pain. However, both treatment and smoking were adjusted for in the final statistical analyses.

In summary, our findings suggest that the development of chronic lumbar radicular pain may be associated with a high IL1 activity relative to IL1Ra. The combination of *IL1A* rs1800587 T allele and *IL1RN* rs2234677 A allele may increase the risk of a chronic outcome and persistent pain following lumbar disc herniation. However, the combination of *IL1B* rs1143627 T allele and *IL1RN* rs2234677 A allele was not associated with increased pain in these patients.
